# Acute Decompensated Heart Failure Secondary to Left Atrial Myxoma: A Case Report Highlighting Diagnostic Challenges and Multidisciplinary Management

**DOI:** 10.7759/cureus.65177

**Published:** 2024-07-23

**Authors:** Vasileios Leivaditis, Eleftherios T Beltsios, Athanasios Papatriantafyllou, Kostas Grapatsas, Francesk Mulita, Manfred Dahm

**Affiliations:** 1 Department of Cardiothoracic and Vascular Surgery, Westpfalz-Klinikum, Kaiserslautern, DEU; 2 Department of Anesthesiology and Intensive Care Medicine, Hannover Medical School, Hannover, DEU; 3 Department of Surgery, General University Hospital of Patras, Patras, GRC

**Keywords:** tricuspid valve repair, mitral valve replacement, extracorporeal life support (ecls), cardiac surgery, mitral valve obstruction, acute decompensated heart failure, heart tumors, left atrial myxoma, cardiac myxoma

## Abstract

Cardiac myxomas are the most common benign tumors of the heart, with clinical manifestations varying significantly based on tumor size. Symptoms can range from asymptomatic and mild non-specific presentations to severe obstructive cardiac and systemic findings. This case report describes a 68-year-old female patient who presented with acute decompensated heart failure. Diagnostic evaluation revealed a left atrial myxoma causing significant mitral valve obstruction. The patient underwent emergency cardiac surgery for tumor removal, complicated by severe mitral and tricuspid valve regurgitation. Following valve replacement and repair, the patient required extracorporeal life support. Despite these complexities, she achieved significant recovery and was discharged in good condition. At follow-up, she remained asymptomatic with no signs of cardiac decompensation. This case highlights the importance of considering cardiac myxoma as a differential diagnosis in such cases to prevent potential complications.

## Introduction

Cardiac myxomas are the most common primary heart tumors in adults, with an incidence of up to 0.2% in autopsy series. Myxomas are more common in women than men, with a 2:1 female-to-male ratio, and can occur at any age [1]. These benign tumors originate from multipotent mesenchyme and typically appear as undifferentiated atrial masses, often pedunculated and attached to the fossa ovalis on the left atrial septum [2,3]. Epidemiologically, myxomas have also been classified into two certain epidemiological types: familial and sporadic. About 75% of myxomas are located in the left atrium, 23% in the right atrium, and 2% in the ventricles. Rarely, they can be present in multiple chambers. These tumors most commonly range in size from 1 to 15 cm in diameter.

Symptoms vary widely, from mild, non-specific symptoms to severe conditions, such as heart failure, stroke, and sudden death. The classic symptom triad includes constitutional, embolic, and obstructive or cardiac manifestations. Due to the vague nature of these symptoms, diagnosis can be challenging, requiring a high level of clinical suspicion [2,4]. Echocardiography is the first-line imaging technique for diagnosis, although it presents limitations. CT scans or cardiac MRI may be needed for detailed evaluation. Histopathological assessment confirms the diagnosis [2].

Timely surgical resection greatly improves prognosis, with postsurgical survival rates similar to the general population. However, tumor recurrences can occur, often due to incomplete resection [3]. This report details the presentation, diagnosis, surgical intervention, and recovery of a patient with a left atrial myxoma causing acute decompensated heart failure.

## Case presentation

Patient background

A 68-year-old female patient with a medical history of hypertension, dyslipidemia, and hypothyroidism presented to the emergency department with severe worsening dyspnea and bilateral peripheral edema. Physical examination revealed signs consistent with acute decompensated heart failure, including jugular venous distention, pulmonary rales, and peripheral edema.

Diagnostic workup

**Figure 1:**
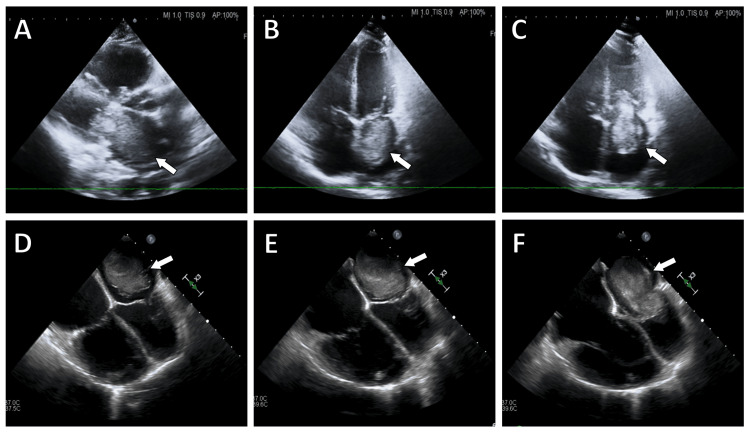
Echocardiographic images showing the left atrial myxoma. Echocardiographic images showing the left atrial myxoma (arrow). Transthoracic echocardiography (TTE) short-axis view (A) and four-chamber view in the systolic (B) and diastolic (C) phases. Transesophageal echocardiography (TEE) images demonstrating the movement of the myxoma in the systolic (D, E) and diastolic phases (E).

Initial laboratory tests indicated elevated B-type natriuretic peptide (BNP) levels (Table 1). An urgent echocardiogram revealed a large left atrial mass with a pedunculated attachment to the interatrial septum, prolapsing into the mitral valve orifice during diastole, causing functional mitral valve stenosis (Figure 1). Coronary angiography did not show any significant coronary artery disease.

**Table 1:** Peoperative vital and laboratory values of the patient. SpO_2_ = oxygen saturation, CRP = C-reactive protein, BUN = blood urea nitrogen, CPK = creatine phosphokinase, CK-MB = MB isoenzyme of CPK, NT-proBNP = N-terminal pro b-type natriuretic peptide, LDH = lactate dehydrogenase, GOT = glutamic-oxaloacetic transaminase, AST = aspartate aminotransferase, GPT = glutamic-pyruvic transaminase, ALT = alanine aminotransferase, pH = potential of hydrogen

Parameter	Obtained value	Reference ranges	International units
Blood pressure	136/92	120/80	mmHg
SpO_2_	92	95-98.5	%
Respiratory rate	21	12-18	Breaths per minute
Pulse rate	122	60-100	Beats per minute
Temperature	37.2	36.5–37.5	°C
White blood cell count	14.26	3.8-10.3	1000/µL
Hemoglobin	11.7	13.5-17.8	g/dL
CRP	73	<5	mg/L
Procalcitonin	0.34	<0.05	ng/mL
Creatinin	1.37	0.7-1.2	mg/dL
BUN	67	8-23	mg/dL
Troponin T	362	0-14	pg/mL
CPK	196	20-200	U/L
CK-MB	11	<25	U/L
NT-proBNP	1243	<386	pg/mL
LDH	291	<250	U/L
GOT (AST)	67	10-50	U/L
GPT (ALT)	94	10-50	U/L
Sodium	143	136-145	mmlol/L
Potassium	3.9	3.4-4.5	mmlol/L
Lactic acid	2.8	0.5-2.2	mmol/L
pH	7.38	7.37-7.45	

Surgical intervention

**Figure 2:**
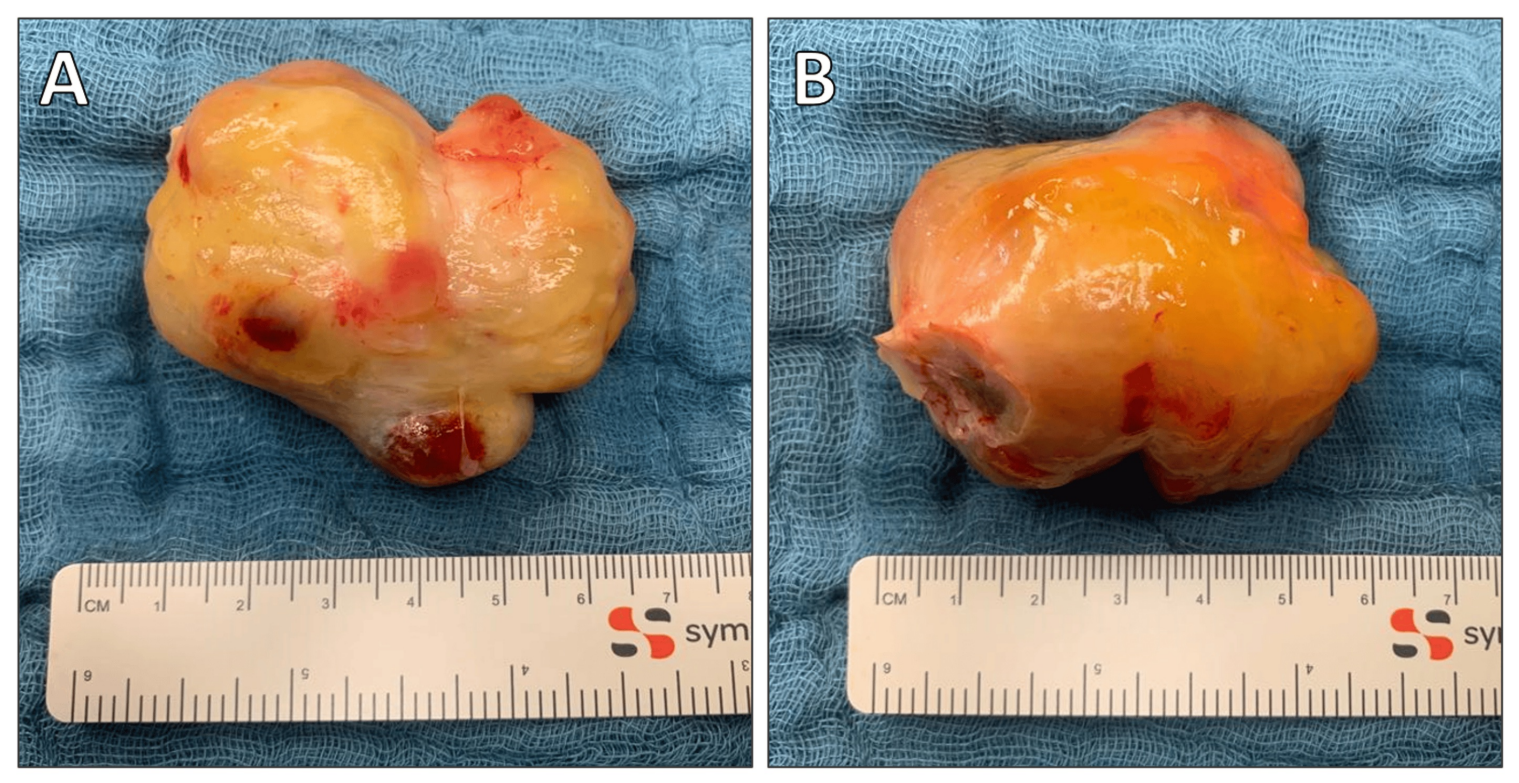
Intraoperative image of the left atrial myxoma after its excision.

**Figure 3:**
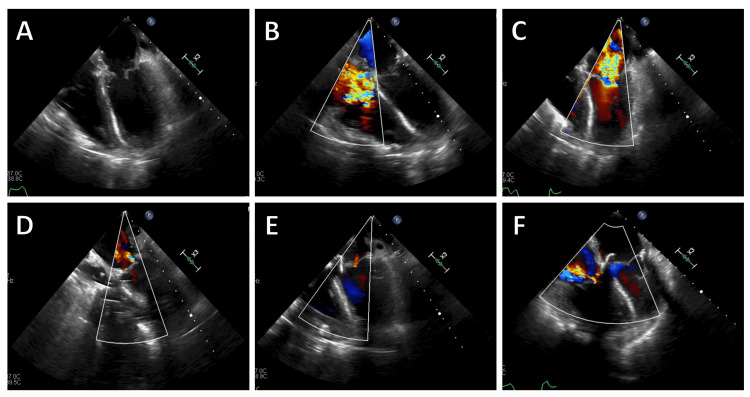
Intraoperative transesophageal echocardiography (TEE) images. (A) After removal of the myxoma, (B) severe tricuspid regurgitation, (C) severe mitral regurgitation, (D) persistent significant regurgitation after mitral valve repair, (E) result after mitral valve replacement, (F) residual less significant tricuspid regurgitation after tricuspid valve annuloplasty.

Due to the acute obstruction caused by the left atrial myxoma, the patient was referred for emergency cardiac surgery. The operation involved complete cardiopulmonary bypass and removal of the myxoma (Figure 2) through the right atrium. The cardiopulmonary bypass time was 156 minutes, and the total aortic cross-clamp time was 92 minutes. Intraoperatively, severe mitral and tricuspid valve regurgitation was noted during weaning from cardiopulmonary bypass, likely masked preoperatively by the myxoma (Figure 3). Attempts at mitral valve repair were unsuccessful, necessitating replacement with a biological prosthesis and tricuspid valve repair using the DeVega annuloplasty technique.

Postoperative course

The patient experienced severe right heart failure and problematic ventilation, requiring extracorporeal life support via the right femoral vein and ascending aorta. Despite aggressive management, the patient exhibited prolonged ventilator dependence, which prompted the decision to perform a tracheotomy on the seventh postoperative day. The tracheotomy facilitated prolonged mechanical ventilation, enhanced pulmonary hygiene, and improved patient comfort. Gradual weaning from extracorporeal support was achieved in the ICU over the following weeks. Continuous echocardiographic monitoring showed significant improvement in cardiac function. By the 11th postoperative day, the patient was successfully weaned from ECLS, and by the 15th postoperative day, she was gradually weaned off the ventilator. Histopathological examination confirmed the diagnosis of cardiac myxoma. Follow-up echocardiography showed significant improvement in cardiac function. The patient was transferred to the regular ward on the 17th postoperative day, where she continued to show marked improvement. Physical therapy and rehabilitation were initiated to enhance her recovery.

Outcome and follow-up

The patient was discharged ambulatory and asymptomatic on the 28th postoperative day. Follow-up visits at one and six months post-surgery revealed an asymptomatic patient with no signs of cardiac decompensation or recurrent myxoma.

## Discussion

Atrial myxomas, the most common benign cardiac tumors, often present with symptoms similar to mitral valve disease or embolic phenomena [5]. This case highlights the need to consider cardiac myxomas in the differential diagnosis of acute decompensated heart failure. The patient’s rapid deterioration required immediate surgical intervention, showcasing the challenges in managing severe valvular regurgitation. Extracorporeal life support postoperatively was crucial for her recovery.

An accurate diagnosis of cardiac myxomas may be challenging with various confounding factors delaying it. Despite their rarity, with an incidence of 0.5 per million annually, they can present with nonspecific symptoms like syncope, dyspnea, or positional breathlessness [4,6,7]. In this case, the patient’s acute heart failure symptoms were due to the myxoma’s obstruction of the mitral valve, mimicking severe mitral stenosis and hiding severe mitral valve regurgitation. This underlines the importance of advanced imaging and timely surgery.

Due to their multifaceted nonspecific clinical presentation that varies from asymptomatic to life-threatening conditions like obstruction, embolization, and stroke [8], myxomas are initially suspected in only 5.7% of patients. Left atrial tumors pose a risk of systemic embolization, while their position can obstruct blood flow, mimicking mitral stenosis and leading to heart failure or sudden death if untreated [9]. In the present case, the large-sized myxoma caused significant obstruction, necessitating urgent surgery.

Depending on their location and morphology, cardiac tumors can produce four main types of clinical manifestations: (i) systemic-constitutional, (ii) embolic, (iii) cardiac, and (iv) secondary metastatic effects [10]. Cardiovascular symptoms such as chest pain, syncope, dyspnea, and angina occur in about 67% of patients, with 28% presenting acute decompensated heart failure. Constitutional symptoms affect 34%, and embolic symptoms are present in 29% of these patients [1,10]. Left-sided myxomas often cause systemic embolization, while right-sided tumors typically embolize to the pulmonary circulation. Pleural effusion is an unusual manifestation and has been reported in a few case studies [5,11]. The increase in LA pressure may cause transudative pleural effusion due to the mitral valve obstruction [5]. Asymptomatic myxomas are often discovered incidentally on imaging [1].

Sudden cardiac death amongst myxoma patients is rare, occurring in about 0.005-0.01% of all sudden deaths, often due to severe acute disturbances in hemodynamics or coronary embolization [1]. This highlights the need for a high index of suspicion in cases with unexplained symptoms.

EKG findings can be nonspecific in 20-40% of patients. The most common EKG finding is left atrial hypertrophy [6]. Echocardiography, both transthoracic (TTE) and transesophageal (TEE), is essential for visualizing and better characterizing cardiac tumors [4]. TEE offers superior image resolution; it is crucial for accurate diagnosis and detailed evaluation and is therefore considered the gold standard for investigating cardiac tumors. It provides crucial information regarding the size, location, and attachment points of tumors, which are critical for planning appropriate surgical interventions and ensuring optimal patient outcomes [4,12]. Crucial factors include the type and location of attachment (whether sessile or pedunculated), surface features (smooth and bosselated versus irregular and fimbriated), and echotexture (identifying hemorrhagic areas and calcification with varying echotextures, homogeneous versus heterogeneous) [13]. Cardiac MRI and CT may provide additional structural and hemodynamic information, complementing the echocardiographic findings [10,14].

While echocardiography is typically the initial imaging modality for cardiac tumors, it can be challenging to distinguish myxomas from other cardiac masses, such as thrombi. Cardiac MRI (CMR) has emerged as a superior imaging modality due to its excellent tissue characterization capability, enabling differentiation between myxomas and thrombi [15] Myxomas often appear as well-defined, smooth, oval or lobular lesions with a narrow pedunculated attachment, frequently at the fossa ovalis. They are typically very mobile and can prolapse through the atrioventricular valves during diastole, causing temporary obstruction to blood flow. Myxomas display heterogeneous intermediate T1 and hyperintense T2 signals due to their mixed tissue composition, and they show a characteristic heterogeneous enhancement pattern on gadolinium-enhanced sequences. In contrast, thrombi are usually broad-based, less mobile, and typically show homogeneous hypointense signals on both T1- and T2-weighted sequences, with no enhancement unless highly organized and fibrous. Other cardiac tumors, such as papillary fibroelastomas, are smaller, highly mobile, and often show uniform enhancement on imaging [16-17]. Table 2 summarizes the key imaging features that help differentiate myxomas from thrombi based on cardiac CMR findings.

**Table 2:** Imaging features differentiating cardiac myxomas from thrombi and other cardiac masses Inversion time on LGE (late gadolinium enhancement) is a specific parameter used in cardiac MRI to nullify the signal from healthy myocardium, thus enhancing the visibility of abnormal tissues. MI = myocardial infarction. References:  [15-17]

Feature	Myxoma	Thrombus	Other tumors (e.g., fibroelastoma)
Location	Left atrium, typically at fossa ovalis	Left atrium (appendage), left ventricle (post-MI)	Valvular endocardium or ventricular outflow tract
Attachment	Narrow, often pedunculated	Broad-based	Narrow, with a pedicle
Mobility	Highly mobile, may prolapse through AV valve	Less mobile	Highly mobile
Shape	Lobulated, irregular	Smooth, more uniform	Small, often round or oval
Size	Variable, can be large (up to 15 cm)	Typically smaller, variable	Generally small (<1 cm)
Echotexture	Heterogeneous	Homogeneous	Homogeneous
Borders	Well-defined	Less well-defined	Well-defined
Functional Impact	Can obstruct AV valve during diastole	May impair ventricular function	Rarely obstructive, more likely embolic
T1 Signal	Heterogeneous intermediate	Homogeneous hypointense	Homogeneous low
T2 Signal	Heterogeneous hyperintense	Homogeneous hypointense	Homogeneous high
Gadolinium Enhancement	Heterogeneous, variable	Typically no enhancement, unless organized	Uniform enhancement
Inversion Time on LGE	200–300 ms	550–650 ms	N/A
Perfusion	Delayed perfusion, heterogeneous uptake	No perfusion	Homogeneous uptake
Calcification	Possible, seen in some cases	Rare, unless chronic	Rare
Hemorrhage	Possible, seen in some cases	Rare	Rare
Consistency	Soft, gelatinous	Firm, organized if chronic	Firm

Surgical removal is the primary treatment for atrial myxomas, aiming for complete excision to restore cardiac function. Post-surgery, patients typically experience symptom resolution. Although recurrence is reported to be relatively rare. with rates raging 2-13%, follow-up with ongoing monitoring is necessary, especially in cases with a family history or incomplete excision [6]. Effective management involves a multidisciplinary approach, integrating cardiologists, cardiothoracic surgeons, radiologists, and pathologists, with providing adequate information to the patient regarding the condition and surgery rationale [4,18].

In summary, this case demonstrates the complexity of the diagnosis and management of atrial myxomas, especially when they present with severe symptoms mimicking or hiding other cardiac conditions. In our case, an underlying severe mitral valve regurgitation was masked through the presence of the myxoma. Advanced imaging and prompt surgical intervention are essential for favorable outcomes.

## Conclusions

This case report underlines the critical importance of considering cardiac myxomas in the differential diagnosis of acute decompensated heart failure. Early diagnosis and prompt surgical intervention are crucial for favorable outcomes in patients with cardiac myxomas. This demonstrates that tailored management strategies are essential for addressing the unique challenges posed by these tumors.
